# Defining the *p*-factor: an empirical test of five leading theories

**DOI:** 10.1017/S0033291722001635

**Published:** 2023-05

**Authors:** Matthew W. Southward, Jennifer S. Cheavens, Emil F. Coccaro

**Affiliations:** 1 Department of Psychology, University of Kentucky, Lexington, KY, USA; 2 Department of Psychology, The Ohio State University, Columbus, OH, USA; 3 Department of Psychiatry and Behavioral Health, The Ohio State University Wexner Medical Center, Columbus, OH, USA

**Keywords:** General factor of psychopathology, impairment, impulsivity, neuroticism, *p*-factor

## Abstract

**Background:**

Despite statistical evidence of a general factor of psychopathology (i.e., *p*-factor), there is little agreement about what the *p*-factor represents. Researchers have proposed five theories: dispositional negative emotionality (neuroticism), impulsive responsivity to emotions (impulsivity), thought dysfunction, low cognitive functioning, and impairment. These theories have primarily been inferred from patterns of loadings of diagnoses on *p*-factors with different sets of diagnoses included in different studies. Researchers who have directly examined these theories of *p* have examined a subset of the theories in any single sample, limiting the ability to compare the size of their associations with a *p*-factor.

**Methods:**

In a sample of adults (*N* = 1833, *M*_age_ = 34.20, 54.4% female, 53.3% white) who completed diagnostic assessments, self-report measures, and cognitive tests, we evaluated statistical *p*-factor structures across modeling approaches and compared the strength of associations among the *p*-factor and indicators of each of these five theories.

**Results:**

We found consistent evidence of the *p*-factor's unidimensionality across one-factor and bifactor models. The *p*-factor was most strongly and similarly associated with neuroticism (*r* = .88), impairment (*r* = .88), and impulsivity (*r* = .87), χ^2^(1)s < .15, *p*s > .70, and less strongly associated with thought dysfunction (*r* = .78), χ^2^(1)s > 3.92, *p*s < .05, and cognitive functioning (*r* = −.25), χ^2^(1)s > 189.56, *p*s < .01.

**Conclusions:**

We discuss a tripartite definition of *p* that involves the transaction of impulsive responses to frequent negative emotions leading to impairment that extends and synthesizes previous theories of psychopathology.

One of the most striking and replicable findings in psychiatric epidemiology is the high rate of comorbidity among psychiatric disorders, with up to two-thirds of people who meet criteria for one disorder meeting criteria for a second (Caspi & Moffitt, 2018). This pattern of comorbidity suggests the presence of broader dimensions of psychopathology, such as internalizing (e.g., depressive, anxiety disorders), externalizing (e.g., substance use, antisocial disorders), and thought disorder (e.g., schizophrenia, paranoid personality disorder (PD), bipolar disorder; Kotov et al., 2020; Krueger & Markon, 2006). However, these broader dimensions are themselves relatively highly correlated (*r*s: .33–.85; Caspi et al., 2014; Krueger & Markon, 2006; Lahey et al., 2012; cf. Wright & Simms, 2015). Based on these correlations, researchers proposed that a single overarching dimension, or general factor, of psychopathology may give rise to psychiatric conditions (Caspi & Moffitt, 2018) or provide a more complete model of the general features of psychopathology (Kotov et al., 2017).

Statistical evidence for a general factor of psychopathology, or *p*-factor, encompassing internalizing and externalizing disorders was first provided by Lahey et al. (2012) and extended by Caspi et al. (2014) to also include psychosis. These studies generated substantial interest in verifying statistical *p*-factors across samples, timeframes, and measures (Smith, Atkinson, Davis, Riley, & Oltmanns, 2020), culminating in a recent meta-analytic factor analysis in which all specific diagnoses demonstrated loadings between .30 and .70 on a *p*-factor (Ringwald, Forbes, & Wright, 2021).

Despite this relatively consistent evidence demonstrating the existence of statistical *p*-factors, there remains little agreement about what exactly these *p*-factors represent (Fried, Greene, & Eaton, 2021). Some researchers have argued that the *p*-factor is a statistical, rather than a substantive, construct resulting from positively correlated components (i.e., a positive manifold; van Bork, Epskamp, Rhemtulla, Borsboom, & van der Maas, 2017) without a strong theoretical account of how it is related to psychopathology (Fried, 2020; Murray, Eisner, & Ribeaud, 2016). Moving to a substantive understanding of *p* requires tests of discriminant validity between falsifiable theories of what the *p*-factor is and is not related to (Fried, 2020). However, few plausible candidates can adequately characterize such a broad construct. Smith et al. (2020) identified four substantive theories of *p* (i.e., dispositional negative emotionality, impulsive responsivity to emotions, thought dysfunction, low cognitive functioning) and proposed a further nonspecific theory (i.e., functional impairment), which we review below.

## Dispositional negative emotionality

Dispositional negative emotionality, or neuroticism, is the tendency to experience frequent and intense negative emotions in response to stressors (Barlow, Sauer-Zavala, Carl, Bullis, & Ellard, 2014). In factor analyses of personality dimensions, neuroticism is often the first factor extracted, explaining the most unique variability among items (Tackett et al., 2013). Neuroticism has demonstrated consistent, medium-to-large-sized associations with mood, anxiety, substance use, eating, psychotic, somatoform, and PDs (Malouff, Thorsteinsson, & Schutte, 2005; Saulsman & Page, 2004) and *p*-factors (*r*s: .40–.99; Brandes, Herzhoff, Smack, & Tackett, 2019; Caspi et al. 2014; Levin-Aspenson, Khoo, & Kotelnikova, 2019). Furthermore, among child and adolescent twins, neuroticism was more strongly related to a *p*-factor than dimensions of prosociality (i.e., empathy and remorse) or daringness (i.e., sensation-seeking and risk-taking) and the genetic component of neuroticism was more strongly related to a *p*-factor than to either internalizing or externalizing dimensions (*r*s: .20–.71; Tackett et al., 2013). Frequent experiences of negative emotions characterize nearly all psychiatric disorders; even in the case of ego-syntonic disorders (e.g., bipolar disorder, anorexia nervosa), frequent experiences of negative emotions may result from interpersonal or functional consequences of behaviors characteristic of the disorder. Thus, it is plausible that the general factor of psychopathology indexes the frequency and intensity of negative emotions.

## Impulsive responsivity to emotions

Alternatively, impulsive, maladaptive responses to negative emotions may define *p* (Carver, Johnson, & Timpano, 2017). Impulsive responses may include impulsive *in*action (e.g., passive avoidance or rumination) or action (e.g., aggressive behaviors), occur without much planning, and be maladaptively overreactive in the context used. *p*-factors have been associated with indicators of impulsivity, such as low conscientiousness (*r* = −.31; Caspi et al., 2014) and poor response inhibition (*r*s: −.34 to −.14; Castellanos-Ryan et al., 2016; Martel et al., 2017). Impulsivity has predicted a range of behaviors characteristic of psychopathology including non-suicidal self-injury (Riley, Combs, Jordan, & Smith, 2015), posttraumatic stress disorder symptoms (Gaher et al., 2014), and substance use (Riley, Rukavina, & Smith, 2016), above and beyond negative emotionality (Settles et al., 2012), suggesting that impulsive responsivity to emotions may be related to *p* regardless of the frequency or intensity of negative emotions.

## Low cognitive functioning

Complementing these affective (i.e., negative emotionality) and behavioral (i.e., impulsivity) theories, some researchers have argued low cognitive functioning best characterizes *p*. *p*-factors have been negatively associated with IQ (*r*s: −.19 to −.10; Caspi et al., 2014; Castellanos-Ryan et al., 2016), executive functioning (*r*s: −.24 to −.07; e.g., attention, processing speed, visual-motor coordination; Castellanos-Ryan et al., 2016; Martel et al., 2017), and positively associated with cognitive problems in everyday life (*r*s: .20–.30; e.g., concentration problems, forgetfulness, difficulties organizing tasks; Caspi & Moffitt, 2018). Low cognitive functioning may predispose people to develop psychopathology because (a) low cognitive functioning indicates neuroanatomical abnormalities that increase a person's risk for developing psychopathology; (b) low cognitive functioning increases the risk and exposure to stressors that increase the likelihood of developing psychopathology; or (c) low cognitive functioning impairs treatment-seeking and -engagement, resulting in an increased burden of psychopathology (Caspi & Moffitt, 2018). However, the magnitude of the relations between Caspi et al.'s (2014) *p*-factor and IQ scores was about half as large as those between the *p*-factor and negative emotionality.

## Thought dysfunction

By contrast, a *p*-factor has been shown to be almost identical to a ‘thought disorder’ factor composed of schizophrenia, mania, and obsessive-compulsive disorder (OCD; *r* = 0.997; Caspi et al., 2014). Thought dysfunction includes ‘illogical, unfiltered, tangential, and reality-distorted and -distorting cognitions’ encompassing delusional beliefs, suicidal thoughts, obsessions, and difficulties making decisions (Caspi & Moffitt, 2018). Thought dysfunction frequently, but not always, demonstrates the highest loading on *p*-factors (*λ*s: .26–.97; Caspi et al. 2014; Laceulle, Vollebergh, & Ormel, 2015; Levin-Aspenson, Watson, Clark, & Zimmerman, 2020; Oltmanns, Smith, Oltmanns, & Widiger, 2018; cf. Forbes et al. 2017; 2021b; Martel *et al*. 2017; Ringwald *et al*. 2021; Stochl *et al*. 2015), and *p*-factors have been uniquely associated with prospective suicide attempts (Hoertel et al., 2015) and manic episodes (Lahey, Krueger, Rathouz, Waldman, & Zald, 2017). Although low cognitive control refers to a lack of resources to make efficient or adaptive decisions, thought dysfunction refers to the degree to which thought patterns correspond with reality. In this theory, *p* indicates how well a person's thought processes align with their environmental context, with more impairing thought processes (e.g., delusions, suicidal thoughts) indicating the highest elevations in *p* (Lahey et al., 2017).

## Impairment

In response to concerns about whether any substantive definition could appropriately capture the range of specific dysfunctions constitutive of psychopathology (e.g., hallucinations, a lack of pleasure, talking excessively), Smith et al. (2020) conceptualize the *p*-factor as an index of impairment (Oltmanns et al., 2018; Widiger & Oltmanns, 2017). Conceptualizing the *p*-factor as impairment resolves how seemingly contrasting responses (e.g., sluggishness vs*.* mania) may positively load onto the same factor because high levels of each symptom can lead to functional impairment (Widiger & Oltmanns, 2017). Lahey et al.'s (2012) *p*-factor, for instance, was more strongly predictive of functionally impairing outcomes (e.g., suicide attempts, psychiatric hospitalization, convictions for violence) than internalizing or externalizing dimensions.

## Limitations

To date, no single study has simultaneously modeled and compared the strength of the relations between a *p*-factor and indicators of these five theories. Thus, researchers are left to compare the strength of associations between different studies with different samples, which may lead to biased conclusions. Furthermore, studies that do include measures of multiple theories have generally included observed correlations with a single indicator of each theory (e.g., Caspi et al., 2014), which may lead to attenuated and less reliable estimates compared to associations among multi-indicator latent variables (Bollen, 1989). Directly comparing these five theories in a single sample would offer the strongest comparative test of the strength of their association with a *p*-factor, providing an initial evaluation of discriminant validity to strengthen the empirical foundations of the theory of the general factor of psychopathology (Fried, 2020).

## Current study

In a secondary data analysis of participants recruited to represent a range of psychopathology with a focus on intermittent explosive disorder, we explored two primary hypotheses. First, we examined three potential factor structures of a *p*-factor using diagnoses, symptom counts, and self-report measures of psychopathology to examine (a) which indicators demonstrated the highest loadings on the *p*-factor and (b) whether the pattern of loadings differed by the factor structure tested. Second, we added indicators of the five theories of *p* to each of these models to compare the strength of the associations between each indicator and the *p*-factor.

## Materials and methods

### Participants

The sample included 1833 community participants (*M*_age_ = 34.20 years, s.d. = 10.73) from the American Midwest. Roughly half of participants identified as female (54.4%; *n* = 997), with a similar number identifying as white (53.3%; *n* = 977). The median reported annual income was $35 000–70 000. A plurality of participants (42.1%; *n* = 772) had earned at least a college degree. Participants were recruited to be in either a clinical or non-clinical group. Inclusion criteria for the clinical group were: being 18 years old or older and meeting criteria for a DSM-5 [American Psychiatric Association (APA), 2013] current or lifetime syndromal (formerly Axis I) or personality disorder. Exclusion criteria involved the presence of a medical illness requiring chronic treatment (e.g., hypertension, heart disease, diabetes, cancer), active mania or substance use disorder, a lifetime diagnosis of a psychotic disorder, or intellectual disability. Inclusion criteria for the non-clinical group involved being 18 years old or older, the absence of a current or lifetime DSM-5 disorder, and the absence of a medical illness requiring chronic treatment. All participants provided informed consent before engaging in study procedures, and the study was approved by the local university Institutional Review Board.

### Measures

#### Indicators of the *p*-factor

##### Diagnostic assessments

Participants completed the Structured Clinical Interview for DSM-IV Axis I Disorders (SCID-I; First, Spitzer, Miriam, & Williams, 2002) to assess mood, anxiety, OCD, stress and trauma-related, eating, bipolar, substance use (including alcohol, cannabis, stimulants, and opioids), intermittent explosive, impulse-control, attention-deficit/hyperactivity (ADHD), conduct, and oppositional defiant disorders. Diagnoses were recorded as present or absent.

Participants also completed the Structured Interview for the Diagnosis of Personality Disorders (SIDP-IV; Pfohl, Blum, & Zimmerman, 1997) to assess PDs. Continuous symptom severity scores (items rated 0–3) were used in all models.

Interviews were conducted by master's or doctoral level clinical psychology students who exhibited good-to-excellent inter-rater reliability (average *κ* = .84; range: .79–.93) across diagnoses of mood, anxiety, substance use, impulse control, and PDs. Because data were originally collected using DSM-IV-TR (APA, 2000) criteria, assessors consulted information gathered during separate clinical interviews with a study psychiatrist and conducted chart reviews to update diagnoses to DSM-5 criteria. Final diagnoses were determined using best-estimate consensus procedures involving research psychiatrists and clinical psychologists (Kosten & Rounsaville, 1992).

##### Self-reported psychopathology.

*Depression:* Participants reported on the intensity of depressive symptoms in the prior 2 weeks using the Beck Depression Inventory-II (21 items rated 0–3; Beck, Steer, & Brown, 1996).

*Anxiety:* Participants reported on the intensity of anxiety symptoms in the prior month using the Beck Anxiety Inventory (21 items rated 0–3; Beck & Steer, 1990).

*Anger:* Participants reported on the intensity and frequency of anger experiences in general using the State-Trait Anger Expression Inventory (10 items rated 0–4; Spielberger, 1999).

*Attention-deficit/hyperactivity:* Participants reported on the current frequency of difficulties with attention and/or hyperactivity using a modified version of the Wender Utah Rating Scale (25 items rated 0–4; Ward, Wender, & Reimherr, 1993).

*Mania:* Participants reported on the intensity of lifetime hypomanic, mixed mania, and depressive symptoms using the General Behavior Inventory-Biphasic subscale (28 items rated 0–3; Depue & Klein, 1988).

*Psychosis:* Participants reported the extent to which they had experienced ideas of reference and ideas of persecution in the past month using the Green et al. Paranoid Thoughts Scale (32 items rated 1–5; Green et al., 2008).

*Trauma:* Participants rated how frequently they had experienced physical, sexual, and emotional abuse and neglect as children and adolescents using the Childhood Trauma Questionnaire-Short Form (28 items rated 1–5; Bernstein et al., 2003).

#### Indicators of the five theories of *p*

*Dispositional negative emotionality:* Participants reported their levels of neuroticism using two subscales from the NEO-Five Factor Inventory (Costa & McCrae, 1992) distinguished by Saucier (1998): Self-Reproach (seven items rated 1–5) and Negative Affect (five items rated 1–5). Participants also completed the Eysenck Personality Questionnaire-Revised-Neuroticism scale (12 items rated 0–2; Eysenck & Eysenck, 1991).

*Impulsive responsivity to emotions:* Participants characterized their impulsive responsivity to emotions using the five subscales of the UPPS-P Impulsive Behavior Scale (59 items rated 1–4; Lynam, Smith, Whiteside, & Cyders, 2006): sensation-seeking, lack of premeditation, lack of perseverance, negative urgency, and positive urgency. Participants also responded to the Barratt Impulsiveness Scale-11 (30 items rated 1–4; Patton, Stanford, & Barratt, 1995) and the Eysenck Personality Questionnaire-Impulsiveness scale (19 items rated 1–3; Eysenck, Pearson, Easting, & Allsopp, 1985).

*Cognitive functioning:* Assessors administered the Wechsler Abbreviated Scale of Intelligence-II (WASI-II; Wechsler, 2011), a brief screen of intelligence consisting of the Vocabulary, Similarities, Block Design, and Matrix Reasoning tests from the Wechsler Adult Intelligence Scale. Responses result in Verbal and Performance IQ scores.

*Thought dysfunction:* Participants reported the degree of general thought dysfunction using the Eysenck Personality Questionnaire-Psychoticism scale (12 items rated 0–2; Eysenck & Eysenck, 1991).

*Impairment:* Assessors who administered the diagnostic assessments documented global assessment of functioning (GAF) scores for each participant (one item rated 0–100; APA, 2013). Lower scores indicate greater impairments in functioning.

### Data analytic method

Little's Missing Completely At Random (MCAR) test suggested the data were not missing completely at random, χ^2^(4433) = 7034.73, *p* < .01. Given the lack of systematic bias in the administration and completion of measures and the small correlations among observed variables and patterns of missingness (*r*s: .05–.20), the data may be considered missing at random (MAR). Thus, we created 100 multiply imputed datasets after 40 000 iterations using Bayesian estimation in Mplus Version 7.0 (Muthén & Muthén, 1998–2012) which asymptotically produces the same estimates as maximum likelihood estimation under MAR. We examined descriptive statistics of the frequency of diagnoses and distributions of continuous variables.

Because models of the *p*-factor have included different combinations of disorders and measures, we first examined the fit of the *p*-factor using confirmatory factor analysis with weighted least square mean and variance adjusted estimation to account for the binary diagnostic data. We examined three solutions to test the stability and generalizability of these models based on previous specifications of the *p*-factor: a one-factor solution and two bifactor solutions.*^1^ In the first bifactor solution, we allowed all items to load onto a higher-order *p*-factor and one of three lower-order factors representing internalizing, externalizing, or thought disorders, which were restricted to be orthogonal to each other and the *p*-factor. In the second bifactor solution, the three lower-order factors were allowed to intercorrelate. Given the current literature on the hierarchical structure of psychopathology (HiTOP; Forbes, 2021; Kotov et al., 2017), we only allowed the somatoform disorder indicator to load onto the *p*-factor and allowed the borderline personality disorder (BPD) indicator to load onto both internalizing and externalizing factors.

Given concerns about the ability of fit indices to accurately distinguish factor analytic models (Bonifay & Cai, 2017; Greene et al., 2019; Stanton et al., 2021), we followed Forbes et al.'s (2021a) recommendations to supplement standard model fit indices [root-mean-square error of approximation (RMSEA; acceptable fit ⩽.10; good fit ⩽ .06; Hu & Bentler, 1999), comparative fit index (CFI) and Tucker–Lewis index (TLI; acceptable fit ⩾.90; excellent fit ⩾.95; Hu & Bentler, 1999), weighted root-mean-square residual (WRMR; good fit <1.00; DiStefano, Lui, Jiang, & Shi, 2017)] with statistics to evaluate the unidimensionality of these models. Because residual variances are not identified with binary indicators, we estimated the reliability of the *p*-factor and lower-order factors with omega hierarchical (*ω*_h_; McDonald, 1999; Zinbarg, Revelle, Yovel, & Li, 2005) using the available continuous symptom count and self-report measures. We use *ω*_h_* to denote omega hierarchical for the *p*-factor and *ω*_h,specific_* to denote omega hierarchical for the lower-order factors to indicate these *ω*_h_'s do not include all variables in the model. *ω*_h_* > .75 indicates sufficient reliability (Reise, Bonifay, & Haviland, 2013a). We calculated explained common variance (ECV; Reise, Scheines, Widaman, & Haviland, 2013b) to assess the proportion of variance across all indicators explained by the *p*-factor relative to the specific factors (ECV > .85 indicates likely unidimensionality; Stucky & Edelen, 2014). We also calculated ECV_S (Forbes et al. 2021a) for each specific factor to estimate the proportion of variance these factors explained. We calculated the percentage of uncontaminated correlations (PUC; Reise et al., 2013b), representing the proportion of correlations that only reflect variance from the *p*-factor (PUC > .70 indicates likely unidimensionality). Finally, we calculated the average parameter bias (APB), representing the difference between item loadings in the one-factor model and the bifactor model (10–15% is deemed acceptable; Muthén, Kaplan, & Hollis, 1987) to assess the similarity of loadings between models.

We then added factors representing the five theories of the *p*-factor to these models. When multiple observed indicators were available (i.e., neuroticism, impulsivity, cognitive functioning), we allowed them to load onto a latent factor to represent the construct. When only single indicators were available (i.e., thought dysfunction, impairment), we created single-indicator latent variables by fixing the residual variance of the indicator to 0. We modeled the covariances between the *p*-factor and factors representing the five theories to test the convergent and discriminant validity of the *p*-factor, while simultaneously modeling the covariances among indicators of the five theories to account for their intercorrelations. We tested for differences in the strength of the absolute value of the standardized associations between the *p*-factor and indicators of each of the five theories using Wald tests. We examined fully standardized results in all models to enhance interpretability. All code is available at https://doi.org/10.17605/osf.io/hs8cp. Because participants did not consent to the open sharing of their data, we provide the raw correlation matrix (Table S1, Online Supplemental Materials), with raw data and measures available upon reasonable request.

## Results

### Descriptive statistics

The most frequently diagnosed conditions were intermittent explosive disorder (30.8%), alcohol use disorder (21.2%), any anxiety disorder (17.9%), and BPD (16.2%; Table 1). Mean scores on self-report measures of depression (Roelofs et al., 2013), anxiety (Gillis, Haaga, & Ford, 1995), trauma (Bernstein et al., 2003), anger (Spielberger, 1999), ADHD (Ward et al., 1993), mania (Chmielewski, Fernandes, Yee, & Miller, 1995), and psychosis (Green et al., 2008) were in line with community norms.

**Table 1.** Descriptive statistics for primary observed indicators

Variable	*n*	%	*M*	s.d.	*α*	Min	Max	Observations (% present)
*Internalizing*								
Diagnoses								
Anxiety disorder	329	17.9	–	–	–	0	1	1833 (100%)
Borderline PD^a^	297	16.2	4.88	5.85	–	0	27	1793 (97.8%)
Depressive disorder	242	13.2	–	–	–	0	1	1833 (100%)
Stress or trauma disorder	174	9.5	–	–	–	0	1	1833 (100%)
Avoidant PD	136	7.4	2.13	3.56	–	0	21	1793 (97.8%)
Eating disorder	58	3.2	–	–	–	0	1	1833 (100%)
OCD or related disorder	47	2.6	–	–	–	0	1	1833 (100%)
Dependent PD	19	1.0	1.52	2.53	–	0	19	1793 (97.8%)
Self-report								
BDI-II	–	–	9.74	11.71	.96	0	59	1450 (79.1%)
BAI	–	–	6.38	7.64	.90	0	56	1182 (64.5%)
CTQ-SF	–	–	43.22	16.70	.87	25	113	1023 (55.8%)
*Externalizing*								
Diagnoses								
Intermittent explosive disorder	565	30.8	–	–	–	0	1	1833 (100%)
Alcohol use disorder	388	21.2	–	–	–	0	1	1833 (100%)
Obsessive-compulsive PD	193	10.5	4.35	3.64	–	0	20	1793 (97.8%)
Cannabis use disorder	170	9.3	–	–	–	0	1	1833 (100%)
Antisocial PD	136	7.4	2.14	3.33	–	0	19	1793 (97.8%)
Narcissistic PD	120	6.5	3.14	4.08	–	0	24	1793 (97.8%)
Stimulant use disorder	77	4.2	–	–	–	0	1	1833 (100%)
ADHD	65	3.5	–	–	–	0	1	1832 (99.9%)
Conduct disorder	45	2.5	–	–	–	0	1	1832 (99.9%)
Histrionic PD	33	1.8	2.21	2.89	–	0	20	1793 (97.8%)
Opioid use disorder	27	1.5	–	–	–	0	1	1833 (100%)
Oppositional defiant disorder	17	.9	–	–	–	0	1	1832 (99.9%)
Impulse-control disorder	17	.9	–	–	–	0	1	1833 (100%)
Self-report								
STAXI Trait Anger	–	–	17.27	9.37	.94	0	40	572 (31.2%)
WRS	–	–	10.47	9.58	.93	0	54	860 (46.9%)
*Thought disorder*								
Diagnoses								
Paranoid PD	132	7.2	2.85	3.41	–	0	17	1793 (97.8%)
Bipolar disorder	12	.7	–	–	–	0	1	1832 (99.9%)
Schizotypal PD	10	.5	1.21	2.06	–	0	19	1793 (97.8%)
Schizoid PD	6	.3	.76	1.55	–	0	14	1793 (97.8%)
Self-report								
GBI-Biphasic Subscale	–	–	15.15	14.12	.95	0	75	650 (35.5%)
GPTS-A	–	–	23.50	10.40	.93	16	58	141 (7.7%)
GPTS-B	–	–	22.27	10.83	.95	16	60	141 (7.7%)
Other psychopathology								
Somatoform disorder	16	.9	–	–	–	0	1	1833 (100%)
*Indicators of theories of p*								
Neuroticism/dispositional negative emotionality								
NEO-FFI Self-Reproach	–	–	8.17	6.61	.89	0	27	422 (23.0%)
NEO-FFI Negative Affect	–	–	10.89	4.93	.75	0	20	422 (23.0%)
EPQ-Neuroticism	–	–	10.27	6.95	.89	0	24	835 (45.6%)
Impulsive responsivity to emotions								
UPPS-Negative Urgency	–	–	1.91	.71	.94	1	3.75	104 (5.7%)
UPPS-Premeditation	–	–	1.89	.46	.84	1	2.73	104 (5.7%)
UPPS-Perseverance	–	–	2.03	.63	.73	1	3.00	104 (5.7%)
UPPS-Sensation Seeking	–	–	2.54	.68	.88	1	4.00	104 (5.7%)
UPPS-Positive Urgency	–	–	1.81	.70	.96	1	4.00	104 (5.7%)
BIS-11	–	–	63.15	12.16	.86	26	107	1240 (67.6%)
EPQ-Impulsivity	–	–	37.44	9.26	.89	19	70	824 (45.0%)
Cognitive functioning								
WASI-Verbal IQ (*T* score)	–	–	54.08	10.08	–	20	78	862 (47.0%)
WASI-Performance IQ (*T* score)	–	–	55.26	9.01	–	10	75	862 (47.0%)
Thought dysfunction								
EPQ-Psychoticism	–	–	6.54	4.05	.70	0	24	832 (45.4%)
Impairment								
GAF	–	–	67.90	13.71	–	26	99	1833 (100%)

*α*, Cronbach's alpha; Min, observed minimum score; Max, observed maximum score; OCD, obsessive-compulsive disorder; PD, personality disorder; BDI-II, Beck Depression Inventory-II; BAI, Beck Anxiety Inventory; CTQ-SF, Childhood Trauma Questionnaire-SF; STAXI, State-Trait Anger Expression Inventory-2; WRS, Wender Utah Rating Scale; GBI, General Behavior Inventory; GPTS, Green et al. Paranoid Thoughts Scale; NEO-FFI, NEO Five Factor Inventory; EPQ, Eysenck Personality Questionnaire; BIS, Barratt Impulsiveness Scale; WASI, Wechsler Abbreviated Intelligence Scale; GAF, global assessment of functioning.

a Borderline PD used as an indicator for internalizing and externalizing disorders.

### Comparing models of the *p*-factor

A one-factor solution of the *p*-factor demonstrated relatively poor fit across imputed datasets, χ^2^(527) = 3813.40, RMSEA = .058, CFI = .857, TLI = .848, WRMR = 2.643, ω* = .92, although all but two indicators demonstrated loadings ⩾.35 (Table 2). The highest loading indicators were BPD symptoms, paranoid PD symptoms, and mania (Table 2).

**Table 2.** Fully standardized loadings of indicators on three models of the *p*-factor

	One-factor	Bifactor, uncorrelated lower-order factors				Bifactor, correlated lower-order factors			
	*p*	*p*	Int	Ext	TD	*p*	Int	Ext	TD
Indicator	*λ* (s.e.)	*λ* (s.e.)	*λ* (s.e.)	*λ* (s.e.)	*λ* (s.e.)	*λ* (s.e.)	*λ* (s.e.)	*λ* (s.e.)	*λ* (s.e.)
Borderline PD	.87 (.01)	.84 (.01)	.15 (.03)	.15 (.03)		.84 (.01)	.18 (.04)	.14 (.05)	
Depression	.70 (.02)	.67 (.02)	.27 (.04)			.68 (.03)	.24 (.07)		
Trauma	.62 (.02)	.60 (.02)	.18 (.05)			.62 (.02)	*.09* (*.07*)		
Anxiety	.65 (.02)	.60 (.02)	.36(.03)			.61 (.03)	.28 (.06)		
Depression Dx	.66 (.02)	.59 (.03)	.43 (.05)			.61 (.04)	.33 (.08)		
Anxiety Dx	.56 (.03)	.49 (.03)	.48 (.05)			.51 (.04)	.35 (.06)		
Dependent PD	.55 (.02)	.51 (.02)	.29 (.04)			.51 (.03)	.35 (.04)		
Trauma/Stress Dx	.52 (.03)	.48 (.03)	.26 (.06)			.51 (.04)	*.11* (*.07*)		
Eating Dx	.52 (.04)	.43 (.05)	.50 (.07)			.47 (.05)	.30 (.09)		
Avoidant PD	.55 (.02)	.50 (.02)	.39 (.04)			.47 (.04)	.65 (.07)		
OCD Dx	.50 (.05)	.44 (.05)	.35 (.08)			.47 (.05)	.19 (.08)		
ADHD	.71 (.02)	.72 (.02)		*.02* (*.04*)		.73 (.02)		*−.09* (*.06*)	
Anger	.66 (.02)	.64 (.03)		.31 (.05)		.67 (.03)		.25 (.07)	
Narcissistic PD	.63 (.02)	.62 (.02)		.17 (.04)		.64 (.02)		*.13* (*.07*)	
Antisocial PD	.64 (.01)	.61 (.02)		.31 (.03)		.63 (.03)		.28 (.06)	
Intermittent Explosive Dx	.63 (.02)	.58 (.03)		.47 (.04)		.61 (.04)		.42 (.06)	
Histrionic PD	.59 (.02)	.59 (.02)		.11 (.04)		.60 (.02)		*.06* (*.06*)	
Obsessive-Compulsive PD	.55 (.02)	.55 (.02)		*.06* (*.04*)		.56 (.02)		*−.01* (*.07*)	
ADHD Dx	.45 (.04)	.44 (.05)		*.12* (*.07*)		.45 (.05)		*.05* (*.07*)	
Impulse Control Dx	.44 (.07)	.45 (.08)		*<.01* (*.08*)		.45 (.08)		*−.03* (*.09*)	
Oppositional Defiant Dx	.45 (.06)	.43 (.07)		*.20* (*.12*)		.45 (.07)		*.20* (*.12*)	
Conduct Dx	.40 (.05)	.36 (.06)		.36 (.07)		.38 (.06)		.37 (.08)	
Stimulant Use Dx	.42 (.04)	.27 (.05)		.83 (.04)		.32 (.07)		.80 (.05)	
Opioid Use Dx	.37 (.06)	.27 (.06)		.56 (.06)		.31 (.07)		.52 (.07)	
Cannabis Use Dx	.38 (.04)	.26 (.04)		.73 (.04)		.31 (.06)		.70 (.05)	
Alcohol Use Dx	.34 (.03)	.24 (.04)		.69 (.04)		.29 (.06)		.66 (.04)	
Mania	.78 (.01)	.83 (.02)			−.16 (.05)	.81 (.02)			*−.03* (*.07*)
Paranoid PD	.79 (.01)	.80 (.01)			.19 (.02)	.77 (.02)			.24 (.04)
Ideas of Persecution	.69 (.04)	.74 (.04)			*−.14* (*.10*)	.72 (.05)			*−.01* (*.15*)
Ideas of Reference	.64 (.05)	.68 (.05)			*−.13* (*.10*)	.65 (.06)			*.04* (*.20*)
Schizotypal PD	.60 (.02)	.63 (.01)			.66 (.12)	.57 (.02)			.52 (.12)
Schizoid PD	.35 (.02)	.34 (.02)			.38 (.07)	.28 (.03)			.60 (.15)
Bipolar Dx	.22 (.08)	.23 (.08)			*.06* (*.10*)	.25 (.09)			*−.12* (*.18*)
Somatoform Dx	.44 (.06)	.45 (.06)				.45 (.06)			

Int, internalizing; Ext, externalizing; TD, thought disorder; PD, personality disorder; Depression, Beck Depression Inventory-II; Trauma, Childhood Trauma Questionnaire-Short Form; Anxiety, Beck Anxiety Inventory; Dx, disorder; OCD, obsessive-compulsive disorder; ADHD, Wender Utah Rating Scale; Anger, State-Trait Anger Expression Inventory-2; Mania, General Behavior Inventory–Biphasic subscale; Ideas of Persecution, Green et al. Paranoid Thoughts Scale-Form B; Ideas of Reference, Green et al. Paranoid Thoughts Scale-Form A.

*Note*. All loadings significant, *p*s < .05, except for those in italics. Indicators ordered largest to smallest by their loading on *p* in the bifactor, correlated lower-order factors model.

By contrast, a bifactor solution of the *p*-factor with orthogonal lower-order factors demonstrated acceptable-to-good fit, χ^2^(493) = 2314.02, RMSEA = .045, CFI = .921, TLI = .910, WRMR = 1.968, with all items loading positively and significantly on the *p*-factor (Table 2).^2^ The *p*-factor was highly reliable, *ω*_h_* = .92, whereas the specific factors were substantially less so, *ω*_h,Internalizing_* = .33, *ω*_h,Externalizing_* = .23, *ω*_h,Thought Disorder_* = .04. The *p*-factor also explained nearly all common variance among all items, ECV = .92, unlike the specific factors: ECV_S_Internalizing_ = .55, ECV_S_Externalizing_ = .24, ECV_S_Thought Disorder_ = .21. Just over 70% of correlations were uncontaminated, PUC = .71, and these loadings were not substantially different from the one-factor model, APB = 5.0%. Again, the highest loading indicators on the *p*-factor were BPD symptoms, mania, and paranoid PD symptoms (Table 2).

Finally, a bifactor solution of the *p*-factor with correlated lower-order factors also demonstrated acceptable-to-good fit, χ^2^(490) = 2238.15, RMSEA = .044, CFI = .924, TLI = .913, WRMR = 1.915, with all items loading positively and significantly on the *p*-factor (Table 2), and *ω*_h_* = .92, *ω*_h,Internalizing_* = .28, *ω*_h,Externalizing_* = .11, *ω*_h,Thought Disorder_* = .04; ECV = .95, ECV_S_Internalizing_ = .63, ECV_S_Externalizing_ = .29, ECV_S_Thought Disorder_ = .09; PUC = .71; and APB = 3.4%, together providing good evidence of unidimensionality.^3^ Again, the highest loading indicators on the *p*-factor were BPD symptoms, mania, and paranoid PD symptoms (Table 2). Lower-order internalizing was negatively associated with externalizing, *r* = −.28, *p* < .01, and positively associated with thought disorder, *r* = .32, *p* < .01; however, externalizing was unrelated to thought disorder, *r* = .05, *p* = .68.

### Testing five theories of *p*

When adding indicators of and factors representing the five theories of *p* to each of the three models of the *p*-factor above, no model demonstrated good fit across indices. We re-fit the models based on theory and modification indices, most notably removing UPPS-Sensation Seeking because it exhibited standardized loadings >1. The bifactor model with correlated lower-order factors was the best-fitting model with acceptable fit by RMSEA, χ^2^(973) = 6253.55, RMSEA = .054, CFI = .848, TLI = .832, WRMR = 2.390, and largely similar loadings on (Δ*λ*s: .01–.11) and associations with (Δ*r*s: .01–.06) the *p*-factor (Tables S4a–S5).

Of the five theory indicators, the *p*-factor was most strongly and similarly associated with impairment (*r* = −.89, *p* < .01), impulsivity (*r* = .87, *p* < .01), and neuroticism (*r* = .86, *p* < .01), χ^2^(1)s < 1.57, *p*s > .20. However, the *p*-factor was more strongly associated with each of these three constructs than with thought dysfunction (*r* = .62, *p* < .01), χ^2^(1)s > 50.41, *p*s < .01, and most weakly associated with cognitive functioning (*r* = −.24, *p* < .01), χ^2^(1)s > 195.92, *p*s < .01.^4^

**Fig. 1.**
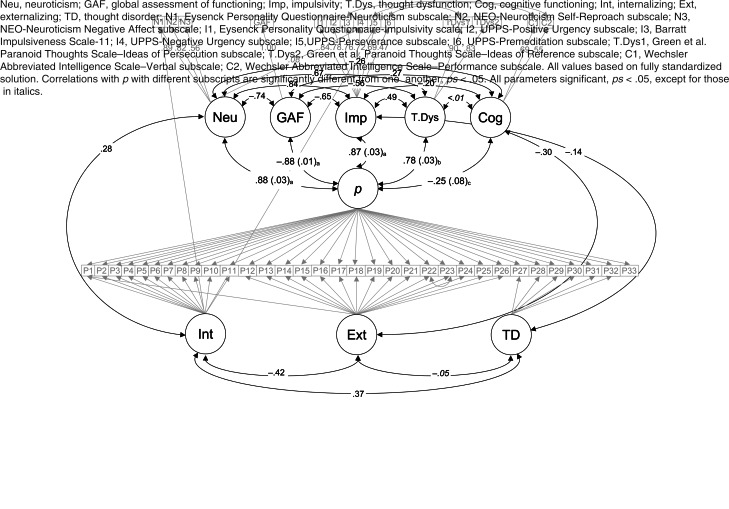
Confirmatory factor analysis comparing the strength of the associations of five theories of *p* with the *p*-factor.

Of note, the EPQ-Psychoticism scale reflects Eysenck's conceptualization of psychoticism as antisocial, creative, egocentric, impulsive, tough-minded, and unempathic (Eysenck, 1987). These characteristics are relatively distinct from Caspi and Moffitt's (2018) conceptualization of thought dysfunction as ‘illogical… and reality-distorted and -distorting cognitions’ with delusional beliefs at the most extreme end of this continuum. Thus, we re-ran the bifactor model of the *p*-factor with correlated lower-order factors, replacing EPQ-Psychoticism as the indicator of thought dysfunction with the two GPTS subscale scores (representing ideas of reference and ideas of persecution), and removing EPQ-Psychoticism based on modification indices. This model demonstrated numerically better fit, χ^2^(926) = 5276.95, RMSEA = .051, CFI = .871, TLI = .856, WRMR = 2.227.^5^ In this model (Fig. 1), the *p*-factor again was most strongly and similarly associated with neuroticism, impairment, and impulsivity, χ^2^(1)s < .20, *p*s > .65. The *p*-factor was more strongly associated with each of these three constructs than with the revised thought dysfunction factor, χ^2^(1)s > 3.92, *p*s < .05, and the weakest association was again with cognitive functioning, χ^2^(1)s > 189.56, *p*s < .01.

## Discussion

In this study, we compared the structure of the *p*-factor among three candidate models in a sample with a range of psychopathology and compared the strength of the associations between the *p*-factor and indicators of five leading theories of *p* to test the convergent and discriminant validity of these theories. Across indices, the *p*-factor was reliable and unidimensional, with similar patterns of factor loadings regardless of model specifications. The *p*-factor was nearly identical to factors representing neuroticism, impulsivity, and impairment, strongly associated with thought dysfunction, and relatively weakly associated with cognitive functioning.

Using Forbes et al.'s (2021a) recommendations, we replicated their findings regarding the high reliability and unidimensionality of the *p*-factor in an independent sample with unique indicators. The consistency of these results across models and samples provides stronger evidence for the existence of a *p*-factor than relying solely on model fit indices (Stanton et al., 2021). The highest loading items on the *p*-factor were BPD, paranoid PD, and mania in line with meta-analytic (Ringwald et al., 2021) and longitudinal (Caspi et al., 2014) research.

However, each diagnostic indicator contains multiple symptoms, so using them to infer the definition of the *p*-factor is less direct and may exhibit more sample-to-sample variability (Levin-Aspenson et al., 2020) than empirically testing the relations between the *p*-factor and specific theories. The *p*-factor was nearly identical to indicators of neuroticism, impulsivity, and impairment. The *p*-factor was also strongly associated with thought dysfunction, but less strongly related to cognitive functioning. These results suggest a model of *p* that extends Barlow et al.'s (2014) model of emotional disorders and synthesizes it with Smith et al.'s (2020) nonspecific impairment interpretation of the *p*-factor. In Barlow et al.'s (2014) model, emotional disorders (e.g., mood disorders, anxiety and related disorders, BPD) are characterized by the transaction between frequent, intense experiences of negative emotions (i.e., neuroticism) and aversive, impulsive reactions to reduce the short-term intensity of those emotions (i.e., impulsivity). However, positive urgency, or the tendency to act rashly in response to positive emotions, demonstrated one of the highest loadings on the impulsivity factor. This suggests the possibility of extending Barlow et al.'s (2014) theory to include impulsive responses to positive emotions that lead to maladaptive consequences, which could in turn prompt frequent negative emotions (i.e., neuroticism). Smith et al.'s (2020) interpretation would add that the transaction between impulsivity and negative and/or positive emotions is necessary but not sufficient to define general psychopathology because it must lead to some level of impairment.

This tripartite definition of the *p*-factor can address Smith et al.'s (2020) challenge that an appropriate definition of the *p*-factor should explain variance in all items loading on the *p*-factor while providing a more falsifiable theory of *p* (Watts, Lane, Bonifay, Steinely, & Meyer, 2020). For instance, hallucinations may only indicate psychopathology if they are hostile or otherwise prompt negative emotions and impulsive attempts to stop them. Strong positive emotions in mania may prompt impulsive and impairing behaviors that may, in turn, lead to negative interpersonal consequences or other dysfunction and thus prompt negative emotions. Restrictive eating behaviors may be an avoidant response to strong negative emotions that can have impairing consequences for a person, despite promoting a temporary feeling of control.

Although we have focused on the relations between the *p*-factor and neuroticism, impulsivity, and impairment, the *p*-factor also demonstrated strong associations with thought dysfunction that varied by the measure of thought dysfunction used. These results, combined with the high loading of paranoid PD symptoms and mania on *p*, suggests the need for further study of the role of thought dysfunction in *p*. In particular, excluding participants with active psychosis and active mania may have restricted the range of thought disorder and thought dysfunction, attenuating the strength of their relations with the *p*-factor. Alternatively, our measures of thought dysfunction may not capture the breadth of Caspi and Moffitt's (2018) definition. We encourage future researchers to include explicit measures of these thought processes in studies of the *p*-factor to more specifically test this theory.

Finally, the relatively small relation between the *p*-factor and cognitive functioning suggests this theory may be less tenable than the others. This finding is in line with Caspi et al. (2014) in which the relation between childhood IQ and the *p*-factor was less than half as large as the relation between the *p*-factor and neuroticism. Cognitive functioning may exert a more distal, developmental effect on the *p*-factor, rather than reflecting psychopathology *per se* (Caspi et al., 2014; Caspi & Moffitt, 2018). Alternatively, method effects may reduce the strength of this association, given that cognitive functioning was measured by a behavioral task whereas indicators of the *p*-factor came from interview assessments and self-reports.

The results of this study should be considered in light of its limitations. Our sample was relatively small compared to other studies of the *p*-factor (e.g., Forbes et al. 2021a), and the exclusion of people with very low cognitive functioning may have reduced the generalizability of our results and attenuated the strength of the relations between the *p*-factor and cognitive functioning. The cross-sectional, between-person nature of the design restricts our ability to draw causal conclusions (Fried, 2020). Although GAF scores characterize functional impairment and are easily implemented in clinical practice, they are only one indicator of impairment that can have low reliability in practice (Vatnaland, Vatnaland, Friis, & Opjordsmoen, 2007). Neither full model of the *p*-factor with indicators of its theories demonstrated good fit across indices, despite the two bifactor models of the *p*-factor alone demonstrating good fit. The high correlations among indicators may suggest this misfit is a result of parsing similar constructs into too many categories (Watts, Boness, Loeffelman, Steinley, & Sher, 2021). Although model fit indices alone may not adequately distinguish models from each other (Greene et al., 2019), relatively low model fit suggests our results should be interpreted cautiously until replicated.

Conceptually, the constructs represented by the theories of *p* may be considered embedded in the diagnostic indicators of the *p*-factor, either through item content or diagnostic criteria, producing circular results. We do not dispute that these constructs are embedded in the diagnostic indicators. However, we note that diagnostic indicators include heterogeneous criteria that vary in how closely they align with the theories of *p* (e.g., most syndromal disorders include an impairment criterion but PDs do not). Rather than indirectly inferring the association between the *p*-factor and theories of *p* based on how heterogeneous diagnostic indicators load onto the *p*-factor, we believe that directly modeling these associations provides a stronger and more straightforward test of these theories, which can contribute to the formalization of theories of *p* by allowing for direct comparisons among these associations. Similarly, the high correlations among indicators may also result from item content overlap. When possible, we excluded scales with direct item overlap (e.g., the NEO-FFI scales include no impulsiveness items). Furthermore, previous researchers have found similarly sized associations between personality and psychopathology factors with and without overlapping items (Walton, Pantoja, & McDermot, 2017). Finally, the tripartite model of *p* we discuss risks defining *p* in terms of the primary characteristics of the lower-order factors. We echo calls from Fried (2020) and others to test these theories in longitudinal studies to examine whether these theories contribute to the development of the *p*-factor (e.g., Williams, Craske, Mineka, & Zinbarg, 2021).

Despite these limitations, we found evidence of a unidimensional *p*-factor in a relatively diverse sample of adults with a range of measures of psychopathology. BPD symptoms, paranoid PD symptoms, and mania loaded most strongly on the *p*-factor regardless of the specific model used. The *p*-factor was most strongly related to neuroticism, impulsivity, and impairment, followed by thought dysfunction, and, to a much lesser degree, low cognitive functioning. We suggest a tripartite definition of the *p*-factor that incorporates transactions between neuroticism and impulsivity leading to impairment, and we encourage future research to test these theories longitudinally.

## Notes

* The notes appear after the main text.

1 We focused on these models because the *p*-factor does not share variance with lower-order factors, making the *p*-factor and associations with it more directly comparable across models (Bornovalova, Choate, Fatimah, Petersen, & Wiernik, 2020; Moore et al., 2020). However, to contextualize our results among alternative conceptualizations of psychopathology, we also examined a correlated factors model and a hierarchical model with individual indicators loading onto specific second-order factors which themselves loaded onto a higher-order *p*-factor.

2 Because multiply imputed models cannot be directly compared in Mplus, we do not report model comparison statistics.

3 A correlated factors model demonstrated worse fit across indices, χ^2^(491) = 3392.27, RMSEA = .057, CFI = .872, TLI = .862, WRMR = 2.481, and very high correlations among all factors: *r*_Internalizing-Externalizing_ = .77, *p* < .01; *r*_Internalizing-Thought Disorder_ = .87, *p* < .01; *r*_Externalizing-Thought Disorder_ = .88, *p* < .01 (Table S2). A hierarchical model demonstrated similarly worse fit across indices, χ^2^(490) = 3376.29, RMSEA = .057, CFI = .873, TLI = .863, WRMR = 2.475, with very high loadings of each specific factor on the *p*-factor: *λ*_Internalizing_ = .87, *p* < .01; *λ*_Externalizing_ = .89, *p* < .01; *λ*_Thought Disorder_ = .97, *p* < .01 (Table S2).

4 Given concerns that some lower-order constructs were overrepresented in the *p*-factor, we re-ran this model including only the three highest-loading diagnoses and three highest-loading self-report measures from each lower-order domain. This model demonstrated relatively poor fit, χ^2^(331) = 5767.68, RMSEA = .095, CFI = .762, TLI = .708, WRMR = 2.413, and nearly identical associations between the *p*-factor and theories of *p*, Δ*r*s: .00–.05 (Figure S1, Online Supplemental Materials).

5 See online Tables S6–S8 for fit statistics, correlations, and item loadings of models with this revised thought dysfunction factor.

## Supplementary material

For supplementary material accompanying this paper visit https://doi.org/10.1017/S0033291722001635. click here to view supplementary material
