# Supplementary material for: Defining the p-factor: an empirical test of five leading theories
Source: Psychol Med. 2022 Jun 17;53(7):2732–43. doi: 10.1017/S0033291722001635 (PMC10235655; doi:10.1017/S0033291722001635)
Supplement: Supplementary file 1 [file S0033291722001635sup001.docx]

Supplemental Online Material

for

**Defining the *p*-Factor: An Empirical Test of Five Leading Theories**

**Table S1**


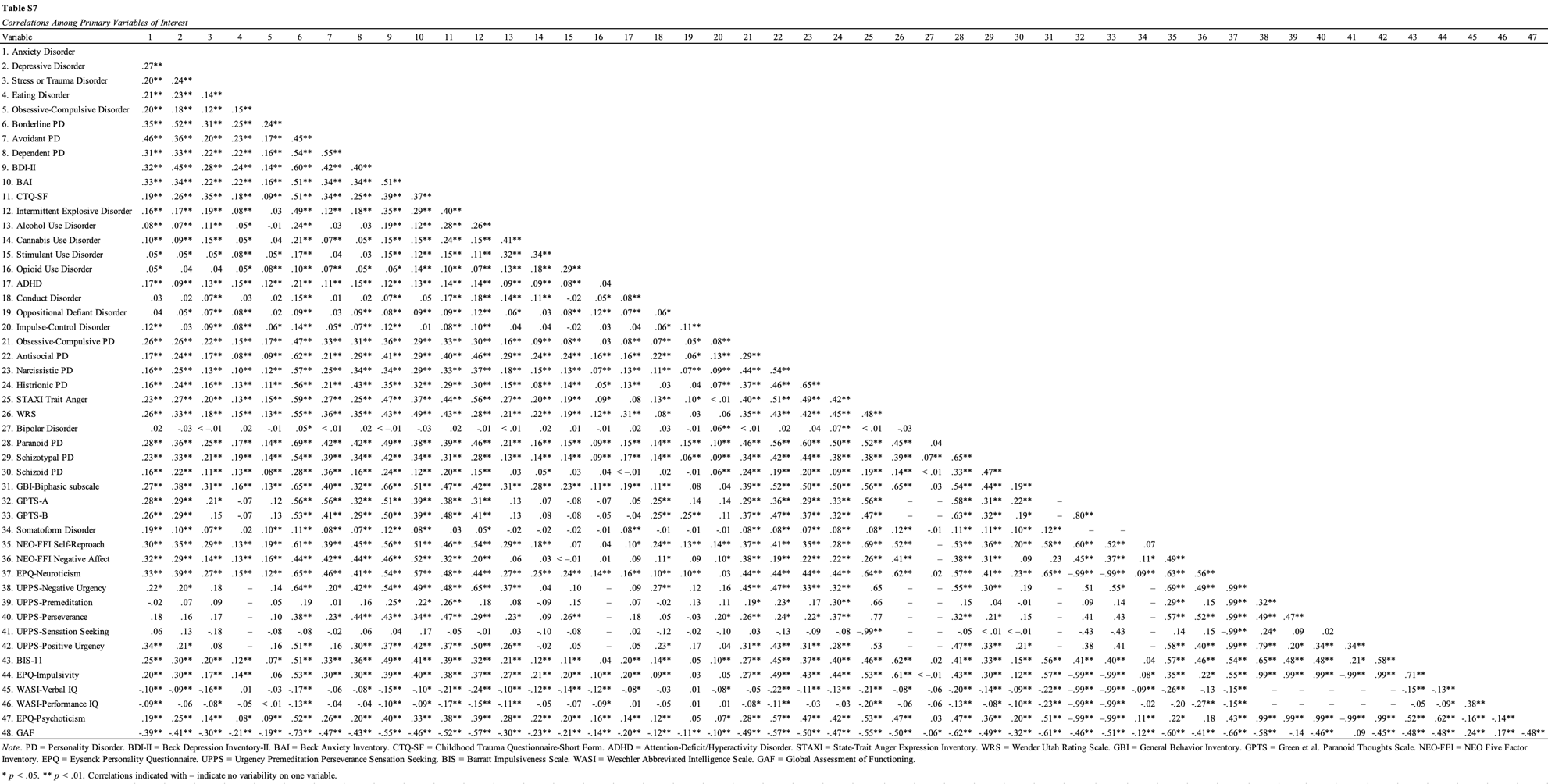


CSV file available at: <https://osf.io/hs8cp/?view_only=18ca2dd08fb74243bb06ffb2a8eaa8fe>

| **Table S2** |  |  |  | |  | |  |  | |  | |
| --- | --- | --- | --- | --- | --- | --- | --- | --- | --- | --- | --- |
| *Fully Standardized Loadings of Indicators on Correlated Factors and Hierarchical Models* | | | | | | | | |  | |  |
|  | Correlated Factors Model | | |  | | Hierarchical Model | | | | |  |
|  | Int | Ext | TD | |  | | Int | Ext | | TD | |
| Indicator | λ (*SE*) | λ (*SE*) | λ (*SE*) | |  | | λ (*SE*) | λ (*SE*) | | λ (*SE*) | |
| Depression | .76 (.02) |  |  | |  | | .76 (.02) |  | |  | |
| Anxiety | .70 (.02) |  |  | |  | | .70 (.02) |  | |  | |
| Depression Dx | .70 (.02) |  |  | |  | | .70 (.02) |  | |  | |
| Trauma | .66 (.02) |  |  | |  | | .67 (.03) |  | |  | |
| Anxiety Dx | .60 (.03) |  |  | |  | | .60 (.03) |  | |  | |
| Avoidant PD | .60 (.02) |  |  | |  | | .60 (.02) |  | |  | |
| Dependent PD | .59 (.02) |  |  | |  | | .59 (.02) |  | |  | |
| Eating Dx | .56 (.04) |  |  | |  | | .56 (.04) |  | |  | |
| Trauma/Stress Dx | .55 (.03) |  |  | |  | | .55 (.03) |  | |  | |
| OCD Dx | .53 (.05) |  |  | |  | | .53 (.05) |  | |  | |
| Borderline PD | .42 (.03) | .51 (.03) |  | |  | | .42 (.03) | .51 (.03) | |  | |
| ADHD |  | .75 (.02) |  | |  | |  | .75 (.02) | |  | |
| Anger |  | .71 (.02) |  | |  | |  | .71 (.02) | |  | |
| Narcissistic PD | | .68 (.02) |  | |  | |  | .68 (.02) | |  | |
| Antisocial PD |  | .68 (.01) |  | |  | |  | .68 (.01) | |  | |
| Intermittent Explosive Dx | | .67 (.02) |  | |  | |  | .69 (.02) | |  | |
| Histrionic PD |  | .63 (.02) |  | |  | |  | .63 (.02) | |  | |
| Obsessive-Compulsive PD | | .58 (.02) |  | |  | |  | .58 (.02) | |  | |
| ADHD Dx |  | .47 (.05) |  | |  | |  | .47 (.05) | |  | |
| Oppositional Defiant Dx | | .46 (.07) |  | |  | |  | .46 (.07) | |  | |
| Stimulant Use Dx | | .46 (.04) |  | |  | |  | .46 (.04) | |  | |
| Impulse Control Dx | | .45 (.08) |  | |  | |  | .45 (.08) | |  | |
| Conduct Dx |  | .43 (.05) |  | |  | |  | .43 (.05) | |  | |
| Cannabis Use Dx | | .41 (.04) |  | |  | |  | .41 (.04) | |  | |
| Opioid Use Dx | | .39 (.06) |  | |  | |  | .39 (.06) | |  | |
| Alcohol Use Dx | | .37 (.03) |  | |  | |  | .37 (.03) | |  | |
| Paranoid PD |  |  | .82 (.02) | |  | |  |  | | .84 (.02) | |
| Mania |  |  | .81 (.02) | |  | |  |  | | .57 (.15) | |
| Ideas of Persecution | |  | .72 (.04) | |  | |  |  | | .73 (.04) | |
| Ideas of Reference | |  | .67 (.05) | |  | |  |  | | .67 (.04) | |
| Schizotypal PD | |  | .63 (.02) | |  | |  |  | | .64 (.03) | |
| Schizoid PD |  |  | .36 (.02) | |  | |  |  | | .37 (.02) | |
| Bipolar Dx |  |  | .19 (.08) | |  | |  |  | | .19 (.08) | |
| *p* | – |  |  | |  | | .87 (.02) | .89 (.02) | | .97 (.03) | |
| Internalizing | – |  |  | |  | |  |  | |  | |
| Externalizing | .77 (.02) | – |  | |  | |  |  | |  | |
| Thought Disorder | .87 (.03) | .88 (.03) | – | |  | |  |  | |  | |
| *Note*. Int = Internalizing. Ext = Externalizing. TD = Thought Disorder. PD = Personality Disorder. Mania = General Behavior Inventory–Biphasic subscale. ADHD = Wender Utah Rating Scale. Ideas of Persecution = Green et al. Paranoid Thoughts Scale-Form B. Anger=State-Trait Anger Expression Inventory-2. Depression=Beck Depression Inventory-II. Dx=Disorder. Ideas of Reference=Green et al. Paranoid Thoughts Scale-Form A. Trauma=Childhood Trauma Questionnaire-Short Form. Anxiety=Beck Anxiety Inventory. OCD=Obsessive-Compulsive Disorder. All loadings significant, *p*s < .05, except those in italics. Indicators ordered largest to smallest by their loading on their respective factor in the correlated factors model. | | | | | | | | | | |  |

| **Table S3** |  |  |  |
| --- | --- | --- | --- |
| *Model Fit Statistics of Three Specifications of the p-Factor with Indicators of Five Theories of p* | | | |
|  | One Factor | Bifactor, Uncorrelated Lower-Order Factors | Bifactor, Correlated Lower-Order Factors |
| χ^2^ (*SD*) | 7255.82 (311.01) | 6606.78 (343.597) | 6253.56 (333.15) |
| *df* | 1011 | 976 | 973 |
| RMSEA (*SD*) | .058 (.001) | .056 (.002) | .054 (.002) |
| CFI (*SD*) | .821 (.009) | .838 (.010) | .848 (.010) |
| TLI (*SD*) | .808 (.010) | .821 (.011) | .832 (.011) |
| WRMR (*SD*) | 2.687 (.071) | 2.494 (.079) | 2.390 (.079) |
| *Note*. Statistics based on 100 multiply imputed datasets using WLSMV estimation. | | | |

| **Table S4a** |  |  |  |  |  |
| --- | --- | --- | --- | --- | --- |
| *Correlations Among p and Indicators of Theories of p in One-Factor Model of p* | | | | | |
|  | 1 | 2 | 3 | 4 | 5 |
| 1. *p* | – |  |  |  |  |
| 2. Neuroticism | .91 (.02)_a_ | – |  |  |  |
| 3. GAF | –.85 (.01)_b_ | –.71 (.02) | – |  |  |
| 4. Impulsivity | .83 (.02)_b_ | .83 (.03) | –.66 (.03) | – |  |
| 5. Thought Dysfunction | .59 (.03)_c_ | .50 (.04) | –.47 (.03) | .74 (.04) | – |
| 6. Cognitive Functioning | –.25 (.06)_d_ | –.24 (.07) | .27 (.04) | –.27 (.07) | –.23 (.05) |
| *Note*. GAF = Global Assessment of Functioning. Statistics based on 100 multiply imputed datasets using WLSMV estimation. All correlations significant, *p*s < .01. Absolute value of correlations with different subscripts are significantly different, *p*s < .05. | | | | | |

| **Table S4b** |  |  |  |  |  |
| --- | --- | --- | --- | --- | --- |
| *Correlations Among p and Indicators of Theories of p in Best-Fitting Bifactor Model of p with Uncorrelated Lower-Order Factors* | | | | | |
|  | 1 | 2 | 3 | 4 | 5 |
| 1. *p* | – |  |  |  |  |
| 2. Neuroticism | .85 (.02)_a_ | – |  |  |  |
| 3. GAF | –.87 (.01)_a_ | –.71 (.02) | – |  |  |
| 4. Impulsivity | .85 (.02)_b_ | .83 (.03) | –.66 (.03) | – |  |
| 5. Thought Dysfunction | .60 (.03)_b_ | .50 (.04) | –.47 (.03) | .70 (.04) | – |
| 6. Cognitive Functioning | –.25 (.06)_c_ | –.24 (.07) | .27 (.04) | –.28 (.07) | –.23 (.05) |
| *Note*. GAF = Global Assessment of Functioning. Statistics based on 100 multiply imputed datasets using WLSMV estimation. All correlations significant, *p*s < .01. Absolute value of correlations with different subscripts are significantly different, *p*s < .05. | | | | | |

| **Table S4c** |  |  |  |  |  |
| --- | --- | --- | --- | --- | --- |
| *Correlations Among p and Indicators of Theories of p in Best-Fitting Bifactor Model of p with Correlated Lower-Order Factors* | | | | | |
|  | 1 | 2 | 3 | 4 | 5 |
| 1. *p* | – |  |  |  |  |
| 2. Neuroticism | .86 (.02)_a_ | – |  |  |  |
| 3. GAF | –.89 (.01)_a_ | –.71 (.02) | – |  |  |
| 4. Impulsivity | .87 (.02)_a_ | .83 (.03) | –.66 (.03) | – |  |
| 5. Thought Dysfunction | .62 (.03)_b_ | .50 (.04) | –.47 (.03) | .70 (.04) | – |
| 6. Cognitive Functioning | –.26 (.06)_c_ | –.24 (.07) | .27 (.04) | –.28 (.07) | –.23 (.05) |
| *Note*. GAF = Global Assessment of Functioning. Statistics based on 100 multiply imputed datasets using WLSMV estimation. All correlations significant, *p*s < .05. Absolute value of correlations with different subscripts are significantly different, *p*s < .05. | | | | | |

| **Table S5** |  |  |  |  |  | |  |  |  |  | | |
| --- | --- | --- | --- | --- | --- | --- | --- | --- | --- | --- | --- | --- |
| *Fully Standardized Loadings of Indicators on Three Models of the p-Factor With Indicators of Five Theories of p Included* | | | | | | | | | |  | | |
|  | One-Factor | Bifactor, Uncorrelated Lower-Order Factors | | | | Bifactor, Correlated Lower-Order Factors | | | | |  |  |
| Indicator | *p* | *p* | Int | Ext | TD | | *p* | Int | Ext | TD | | |
|  | λ (*SE*) | λ (*SE*) | λ (*SE*) | λ (*SE*) | λ (*SE*) | | λ (*SE*) | λ (*SE*) | λ (*SE*) | λ (*SE*) | | |
| Borderline PD | .86 (.01) | .86 (.01) | .12 (.03) | *.01 (.03)* |  | | .83 (.01) | .23 (.04) | *–.02 (.05)* |  | | |
| Depression | .71 (.02) | .68 (.02) | .27 (.03) |  |  | | .65 (.02) | .34 (.03) |  |  | | |
| Trauma | .64 (.02) | .63 (.02) | .13 (.05) |  |  | | .62 (.02) | .15 (.05) |  |  | | |
| Anxiety | .66 (.02) | .61 (.02) | .36 (.03) |  |  | | .59 (.02) | .36 (.04) |  |  | | |
| Depression Dx | .67 (.02) | .62 (.03) | .36 (.04) |  |  | | .59 (.03) | .40 (.05) |  |  | | |
| Obsessive-Compulsive PD | .54 (.02) | .52 (.02) | .13 (.03) | *–.01 (.03)* |  | | .50 (.02) | .18 (.04) | *<–.01 (.05)* |  | | |
| Trauma/Stress Dx | .52 (.03) | .50 (.04) | .18 (.06) |  |  | | .49 (.04) | .18 (.05) |  |  | | |
| Anxiety Dx | .57 (.03) | .51 (.03) | .42 (.05) |  |  | | .48 (.03) | .41 (.04) |  |  | | |
| Dependent PD | .54 (.02) | .50 (.02) | .36 (.04) |  |  | | .47 (.02) | .39 (.04) |  |  | | |
| Eating Dx | .53 (.05) | .47 (.05) | .34 (.07) |  |  | | .46 (.05) | .29 (.06) |  |  | | |
| OCD Dx | .48 (.05) | .44 (.05) | .27 (.08) |  |  | | .43 (.05) | .25 (.07) |  |  | | |
| Avoidant PD | .51 (.02) | .46 (.02) | .49 (.03) |  |  | | .40 (.03) | .66 (.04) |  |  | | |
| ADHD | .76 (.02) | .79 (.02) |  | –.15 (.03) |  | | .77 (.02) |  | .23 (.04) |  | | |
| Anger | .70 (.02) | .70 (.02) |  | .16 (.04) |  | | .72 (.02) |  | –.11 (.05) |  | | |
| Intermittent Explosive Dx | .67 (.02) | .67 (.02) |  | .32 (.05) |  | | .70 (.02) |  | –.32 (.05) |  | | |
| Antisocial PD | .66 (.01) | .66 (.02) |  | .18 (.03) |  | | .68 (.02) |  | –.13 (.05) |  | | |
| Narcissistic PD | .60 (.02) | .61 (.02) |  | *.05 (.03)* |  | | .61 (.02) |  | *.03 (.06)* |  | | |
| Histrionic PD | .57 (.02) | .59 (.02) |  | *–.02 (.03)* |  | | .59 (.02) |  | *.08 (.04)* |  | | |
| ADHD Dx | .49 (.05) | .50 (.05) |  | *.02 (.07)* |  | | .50 (.05) |  | *.01 (.07)* |  | | |
| Impulse Control Dx | .46 (.07) | .47 (.08) |  | *–.06 (.08)* |  | | .46 (.08) |  | *.09 (.09)* |  | | |
| Stimulant Use Dx | .42 (.04) | .38 (.05) |  | .79 (.04) |  | | .45 (.05) |  | –.75 (.05) |  | | |
| Conduct Dx | .42 (.05) | .40 (.05) |  | .31 (.09) |  | | .43 (.05) |  | –.31 (.10) |  | | |
| Oppositional Defiant Dx | .42 (.07) | .39 (.07) |  | .42 (.09) |  | | .43 (.07) |  | –.32 (.11) |  | | |
| Alcohol Use Dx | .36 (.03) | .34 (.03) |  | .65 (.04) |  | | .39 (.04) |  | –.63 (.04) |  | | |
| Cannabis Use Dx | .34 (.04) | .33 (.04) |  | .74 (.04) |  | | .39 (.04) |  | –.66 (.05) |  | | |
| Opioid Use Dx | .38 (.06) | .34 (.06) |  | .56 (.05) |  | | .39 (.07) |  | –.51 (.06) |  | | |
| Bipolar Dx | .21 (.08) | .18 (.09) |  | .38 (.09) | *–.13 (.20)* | | .24 (.10) |  | –.31 (.13) | *–.17 (.18)* | | |
| Mania | .81 (.01) | .81 (.02) |  |  | .20 (.07) | | .79 (.02) |  |  | .21 (.06) | | |
| Paranoid PD | .76 (.01) | .76 (.01) |  |  | .17 (.09) | | .73 (.02) |  |  | .30 (.06) | | |
| Ideas of Persecution | .64 (.04) | .64 (.05) |  |  | .54 (.09) | | .60 (.06) |  |  | .40 (.14) | | |
| Schizotypal PD | .56 (.01) | .57 (.02) |  |  | *.03 (.10)* | | .53 (.02) |  |  | .24 (.09) | | |
| Ideas of Reference | .58 (.05) | .58 (.06) |  |  | .68 (.15) | | .52 (.06) |  |  | .54 (.17) | | |
| Schizoid PD | .30 (.02) | .30 (.02) |  |  | *.07 (.09)* | | .26 (.03) |  |  | .29 (.09) | | |
| Somatoform Dx | .42 (.07) | .42 (.07) |  |  |  | | .43 (.07) |  |  |  | | |
| *Note*. Int = Internalizing. Ext = Externalizing. TD = Thought Disorder. PD = Personality Disorder. ADHD = Attention Deficit-Hyperactivity Disorder. Dx = Disorder. OCD = Obsessive-Compulsive Disorder. All loadings significant, *p*s < .05, except those in italics. Indicators ordered largest to smallest by their loading on *p* in the Bifactor, Correlated Lower-Order Factors model. | | | | | | | | | | | |  |

***Figure S1***

*Confirmatory Factor Analysis Comparing the Strength of the Associations of Five Theories of p With the p-Factor (Restricted Item Set)*

N1

N2

TDys

N3

I5

I4

I1

I2

I3

C1

C2

–.71

–.66

.71

.85

–.47

GAF

.90

.84

.63

.50

1.00

–.24

.27

–.27

–.27 (.06)_c_

.82.77.77.75.68

1.00

.54

.70

–.23

*–.09*

.87 (.03)_a_

.35

–.85 (.01)_a_

.56 (.03)_b_

.91 (.02)_a_

P1

P2

P3

P4

P5

P9

P17

P16

P15

P14

P8

P7

P13

P11

P6

P12

P10

–.74

.25

*.12*

**Figure 1.** Neu = Neuroticism. GAF = Global Assessment of Functioning. Imp = Impulsivity. T.Dys = Thought Dysfunction. Cog

| **Table S6** |  |  |  |
| --- | --- | --- | --- |
| *Model Fit Statistics of Three Specifications of the p-Factor with Indicators of Five Theories of p, Revised Thought Dysfunction* *Factor* | | | |
|  | One Factor | Bifactor, Uncorrelated Lower-Order Factors | Bifactor, Correlated Lower-Order Factors |
| χ^2^ (*SD*) | 6449.68 (326.05) | 5549.48 (298.23) | 5276.95 (277.43) |
| *df* | 964 | 929 | 926 |
| RMSEA (*SD*) | .056 (.002) | .052 (.002) | .051 (.002) |
| CFI (*SD*) | .838 (.010) | .864 (.009) | .871 (.008) |
| TLI (*SD*) | .826 (.010) | .848 (.010) | .856 (.009) |
| WRMR (*SD*) | 2.571 (.078) | 2.311 (.076) | 2.227 (.071) |
| *Note*. Statistics based on 100 multiply imputed datasets using WLSMV estimation. | | | |

| **Table S7a** |  |  |  |  |  |
| --- | --- | --- | --- | --- | --- |
| *Correlations Among p and Indicators of Theories of p in One-Factor Model of p, Revised Thought Dysfunction Factor* | | | | | |
|  | 1 | 2 | 3 | 4 | 5 |
| 1. *p* | – |  |  |  |  |
| 2. Neuroticism | .92 (.02)_a_ | – |  |  |  |
| 3. GAF | –.86 (.01)_a_ | –.71 (.03) | – |  |  |
| 4. Impulsivity | .87 (.03)_a_ | .85 (.06) | –.67 (.03) | – |  |
| 5. Thought Dysfunction | .73 (.04)_b_ | .66 (.06) | –.55 (.05) | .47 (.06) | – |
| 6. Cognitive Functioning | –.27 (.05)_c_ | –.28 (.08) | .27 (.04) | –.22 (.08) | *–.03 (.16)* |
| *Note*. GAF = Global Assessment of Functioning. Statistics based on 100 multiply imputed datasets using WLSMV estimation. All non-italicized correlations significant, *p*s < .05. Absolute value of correlations with different subscripts are significantly different, *p*s < .05. | | | | | |

| **Table S7b** |  |  |  |  |  |
| --- | --- | --- | --- | --- | --- |
| *Correlations Among p and Indicators of Theories of p in Bifactor Model of p with Uncorrelated Lower-Order Factors, Revised Thought Dysfunction Factor* | | | | | |
|  | 1 | 2 | 3 | 4 | 5 |
| 1. *p* | – |  |  |  |  |
| 2. Neuroticism | .89 (.03)_a_ | – |  |  |  |
| 3. GAF | –.88 (.01)_a_ | –.75 (.03) | – |  |  |
| 4. Impulsivity | .89 (.03)_a_ | .88 (.07) | –.66 (.03) | – |  |
| 5. Thought Dysfunction | .78 (.04)_b_ | .66 (.07) | –.56 (.05) | .53 (.07) | – |
| 6. Cognitive Functioning | –.24 (.06)_c_ | –.24 (.08) | .27 (.04) | –.22 (.08) | *<.01 (.16)* |
| *Note*. GAF = Global Assessment of Functioning. Statistics based on 100 multiply imputed datasets using WLSMV estimation. All non-italicized correlations significant, *p*s < .05. Absolute value of correlations with different subscripts are significantly different, *p*s < .05. | | | | | |

| **Table S8** |  |  |  |  |  | |  |  |  |  | |  |
| --- | --- | --- | --- | --- | --- | --- | --- | --- | --- | --- | --- | --- |
| *Fully Standardized Loadings of Indicators on Three Models of the p-Factor With Indicators of Five Theories of p Included, Revised Thought Dysfunction Factor* | | | | | | | | | |  | | |
|  | One-Factor | Bifactor, Uncorrelated Lower-Order Factors | | | | Bifactor, Correlated Lower-Order Factors | | | | |  |  |
| Indicator | *p* | *p* | Int | Ext | TD | | *p* | Int | Ext | TD | |  |
|  | λ (*SE*) | λ (*SE*) | λ (*SE*) | λ (*SE*) | λ (*SE*) | | λ (*SE*) | λ (*SE*) | λ (*SE*) | λ (*SE*) | |  |
| Borderline PD | .86 (.01) | .84 (.01) | .16 (.03) | .05 (.03) |  | | .83 (.01) | .20 (.04) | *.08 (.04)* |  | |  |
| Depression | .71 (.01) | .67 (.02) | .28 (.04) |  |  | | .67 (.02) | .28 (.04) |  |  | |  |
| Trauma | .63 (.02) | .61 (.02) | .17 (.05) |  |  | | .62 (.02) | .13 (.05) |  |  | |  |
| Depression Dx | .67 (.02) | .61 (.03) | .38 (.05) |  |  | | .61 (.03) | .37 (.05) |  |  | |  |
| Anxiety | .66 (.02) | .61 (.02) | .35 (.04) |  |  | | .61 (.02) | .33 (.03) |  |  | |  |
| Anxiety Dx | .57 (.03) | .50 (.03) | .42 (.05) |  |  | | .51 (.03) | .37 (.05) |  |  | |  |
| Dependent PD | .54 (.02) | .50 (.02) | .36 (.04) |  |  | | .50 (.02) | .38 (.04) |  |  | |  |
| Trauma/Stress Dx | .52 (.03) | .49 (.04) | .20 (.06) |  |  | | .50 (.04) | .14 (.06) |  |  | |  |
| Eating Dx | .53 (.05) | .46 (.05) | .37 (.07) |  |  | | .47 (.05) | .31 (.07) |  |  | |  |
| Avoidant PD | .52 (.02) | .45 (.02) | .51 (.04) |  |  | | .44 (.03) | .60 (.04) |  |  | |  |
| OCD Dx | .48 (.05) | .43 (.05) | .26 (.09) |  |  | | .44 (.05) | .21 (.08) |  |  | |  |
| ADHD | .75 (.02) | .78 (.02) |  | –.15 (.03) |  | | .77 (.02) |  | –.22 (.04) |  | |  |
| Anger | .69 (.02) | .68 (.03) |  | .25 (.05) |  | | .70 (.03) |  | .24 (.05) |  | |  |
| Intermittent Explosive Dx | .67 (.02) | .64 (.02) |  | .49 (.05) |  | | .67 (.02) |  | .47 (.04) |  | |  |
| Antisocial PD | .64 (.01) | .63 (.02) |  | .23 (.03) |  | | .65 (.02) |  | .22 (.03) |  | |  |
| Narcissistic PD | .59 (.02) | .59 (.02) |  | .07 (.04) |  | | .60 (.02) |  | *.05 (.04)* |  | |  |
| Histrionic PD | .57 (.02) | .58 (.02) |  | *.01 (.04)* |  | | .58 (.02) |  | *–.03 (.04)* |  | |  |
| Obsessive-Compulsive PD | .53 (.02) | .51 (.02) | .15 (.03) | *.03 (.03)* |  | | .50 (.02) | .21 (.04) | *.08 (.05)* |  | |  |
| ADHD Dx | .49 (.05) | .49 (.05) |  | *.01 (.07)* |  | | .49 (.05) |  | *–.04 (.07)* |  | |  |
| Impulse Control Dx | .46 (.07) | .47 (.08) |  | *.02 (.10)* |  | | .47 (.08) |  | *<–.01 (.10)* |  | |  |
| Stimulant Use Dx | .41 (.04) | .35 (.05) |  | .78 (.05) |  | | .41 (.05) |  | .68 (.07) |  | |  |
| Conduct Dx | .41 (.05) | .38 (.06) |  | .36 (.08) |  | | .41 (.05) |  | .39 (.08) |  | |  |
| Oppositional Defiant Dx | .42 (.07) | .38 (.07) |  | .42 (.09) |  | | .41 (.07) |  | .37 (.09) |  | |  |
| Opioid Use Dx | .38 (.06) | .31 (.06) |  | .56 (.06) |  | | .36 (.06) |  | .45 (.07) |  | |  |
| Cannabis Use Dx | .34 (.04) | .30 (.04) |  | .69 (.05) |  | | .35 (.04) |  | .58 (.06) |  | |  |
| Alcohol Use Dx | .36 (.03) | .31 (.03) |  | .61 (.04) |  | | .35 (.04) |  | .55 (.05) |  | |  |
| Mania | .80 (.01) | .82 (.01) |  |  | –.08 (.04) | | .82 (.01) |  |  | *–.01 (.04)* | |  |
| Paranoid PD | .76 (.01) | .76 (.01) |  | .09 (.03) | .28 (.02) | | .74 (.01) |  | .09 (.03) | .32 (.02) | |  |
| Schizotypal PD | .56 (.01) | .59 (.02) |  |  | .59 (.06) | | .54 (.02) |  |  | .58 (.04) | |  |
| Schizoid PD | .29 (.02) | .32 (.02) |  |  | .47 (.05) | | .28 (.02) |  |  | .54 (.04) | |  |
| Bipolar Dx | .20 (.08) | *.15 (.09)* |  | .40 (.08) | *.11 (.09)* | | .18 (.09) |  | .42 (.09) | *.06 (.10)* | |  |
| Somatoform Dx | .42 (.07) | .42 (.07) |  |  |  | | .42 (.07) |  |  |  | |  |
| *Note*. Int = Internalizing. Ext = Externalizing. TD = Thought Disorder. PD = Personality Disorder. ADHD = Attention Deficit-Hyperactivity Disorder. Dx = Disorder. OCD = Obsessive-Compulsive Disorder. All loadings significant, *p*s < .05, except those in italics. Indicators ordered largest to smallest by their loading on *p* in the Bifactor, Correlated Lower-Order Factors model. | | | | | | | | | | | |  |
